# Students’ “COVID-19” and “school” perceptions in the pandemic

**DOI:** 10.3389/fpsyg.2022.897177

**Published:** 2022-08-08

**Authors:** Ömer F. Vural, Mehmet Başaran, Zeynep Demirtaş, Aynur R. Karamanlı, Cansu Bayrakcı

**Affiliations:** ^1^Department of Education Sciences, Faculty of Education, Sakarya University, Sakarya, Turkey; ^2^Department of Education Sciences, Faculty of Education, Gaziantep University, Gaziantep, Turkey; ^3^Department of Education Sciences, Institute of Education Sciences, Sakarya University, Sakarya, Turkey; ^4^Department of Psychology, Social Sciences Institute, Üsküdar University, Istanbul, Turkey

**Keywords:** education, COVID-19, high school students, metaphor, pandemic (COVID-19)

## Abstract

This study aims to reveal high school students’ perceptions of COVID-19 and schools in the pandemic process through metaphors. In the study, phenomenology research design based on the qualitative research method was used. The study was carried out with the participation of 134 students at all grade levels from high school. The data were analyzed by content analysis. The metaphors were categorized according to their similarities, and their frequency values were calculated. Seventy-six metaphors and eight categories about COVID-19 were reached, and “flu,” “prison,” and “snake” are the metaphors that have the highest frequency values. They explained the metaphors by making associations with the disease, fast-spreading and damaging things. In the COVID-19 process, 78 metaphors and eight categories were found out for the school. Based on the metaphors created by the students, suggestions were made depending on the research results. It was determined that students had negative perceptions about the COVID-19 pandemic and the school in this process, and it was suggested that this could be eliminated again with educational activities.

## Introduction

Pandemic is the spread of a disease or an infectious agent in a very wide area such as countries, continents, and even the whole world (Ministry of Health, 2020). The COVID-19 pandemic emerged in Wuhan, China, approximately 2 years ago, and this pandemic, which could not be prevented from spreading, turned into a serious health problem around the world in a short span of time. The COVID-19 was declared as a pandemic by the World Health Organization (WHO) in March 2020. Besides, this unexpected encounter with the situation outside the usual order has also brought new practices to the agenda. Measures and regulations such as working from home, lockdown periods, curfew restrictions, social distance, and the use of mask have made the living conditions difficult. To prevent the pandemic from spreading and to keep it under control, a decision was taken to close the places that are crowded and may have problems in the implementation of the measures. Schools and universities have also been included in these places.

The coronavirus crisis and the restrictions that come with it (flight bans, lockdown periods, and closure of some workplaces for a while) have caused a lot of negativity, especially in health, economy, psychology, and social life, and also brought new regulations on education. Some health measures have been taken in almost all the countries where the pandemic was seen. In parallel with this, schools were closed and the transition to distance (online) education was made to ensure the continuity and sustainability of education and not to distract students from education (Telli Yamamoto and Altun, 2020). The situation did not differ in Turkey, and face-to-face education was suspended considering the serious threats of the pandemic.

Since it is not predictable when the pandemic process will end, a route has been drawn to ensure continuity in education, to keep students in the education process by providing opportunities, and to minimize disruption in educational programs. This route has started a new era for educators and students. The coronavirus effect required educators and students to adapt to a new process in education as well as social life. Considering the situation, it is thought that it will be effective to evaluate the students’ and educators’ experiences gained in the process and their points of view with the data obtained through metaphors (Bozkurt, 2020).

A great deal of study is available in the literature that was conducted by the use of metaphor method. These studies appear in researches on different branches of social sciences. During the COVID-19 pandemic, metaphor studies were carried out to measure perception on different concepts in the field of education. These studies especially focus on students’ perception of distance education (Bozdaǧ and Dinç, 2020; Bozkurt, 2020). Arslan and Filiz (2020) worked on the evaluation of medical students’ perceptions of the COVID-19 pandemic. It was revealed that the students relied on the measures taken by the state during this process and they were conscious of taking individual precautions and obeying them, and it was concluded that their concerns about the virus were high. Saatçi and Aksu (2020) carried out a study to determine the coronavirus perceptions of undergraduate foreign students through metaphor. When the metaphors used by the students were examined, they evaluated it as a process that the plans of the whole world suddenly changed, health was threatened, people died desperately, freedom was restricted, and people were afraid of each other (Niemi and Kousa, 2020). A case study was made on the perceptions of students and teachers during the COVID pandemic in a high school in Finland, and a research was conducted to link the perception of COVID-19, especially with the educational processes of students. With the transition to distance education due to COVID-19, students stated that they were tired and their motivation was low. It has been revealed that the challenges about the learning method (along with a new process) also stemmed from technological problems. There is no metaphorical study on school perception in the COVID-19 process. However, there are previous studies on school perception in the field. Özdemir (2012), in his study examining the metaphorical school perception of high school students, concluded that freshmen and the ones who had low income thought that school has a protective and supportive side, while sophomores, juniors, seniors, and the ones who had high income found the school as a repressive place. In Özdemir’s (2013) study on school engagement and school perception, it was revealed that students’ perception of school was at a medium level.

There is no metaphorical study examining high school students’ perceptions of the COVID-19 pandemic and school in this process. The purpose of this research is to examine and evaluate high school students’ perceptions of the COVID-19 pandemic and the school in this process through metaphors. In line with the general purpose, the following questions were tried to be answered:

- What are the metaphorical perceptions of high school students toward the COVID-19 pandemic?
- What are the metaphorical perceptions of high school students toward school during the COVID-19 process?

## Method

### Research pattern

The research is a qualitative study that seeks to examine the perceptions of high school students toward the COVID-19 pandemic and the school through metaphors in the process. The use of metaphors of the individual is in comparison with another concept and analogy from common aspects; it is an indication that it is reflected in the perspective and interpretation of the cognitive world. Metaphor, which provides connections and associations between situations, is a mental tool. What is expressed in the metaphors referenced here is very important for the researcher’s evaluations (Arslan and Bayrakçı, 2006; Bozkurt, 2020).

During the COVID-19 pandemic, studies have been carried out on the views of teachers and school administrators on how education is shaped, how students and educators are affected by this process, or how it is reflected in education. Metaphor is an effective method for conducting in-depth research in qualitative research that cannot be done face to face, such as observation and interviewing. A new or complex situation, subject, fact; It is tried to be explained in a more understandable way with metaphor (Güneş and Firat, 2016). The word metaphor contains many structural meanings. “The fact that the concept corresponds to more than one meaning is mainly due to the opening up of an infinite universe of meaning in the sense of ‘transport’ (one meaning to another)” (Güneş and Tezcan, 2017). In the dictionary of the Turkish Language Association (TLA), it metaphorically explained the meaning of the word metaphor. Metaphors are one of the most important mental phenomena that form, regulate, direct, and control our thoughts about the occurrence, process, and shape of an event (Saban, 2004). Metaphors are used to detect and identify people’s perceptions of anything. These perceptions affect how people position any situation or concept in their own world (Kozan and Şahin Zetreoǧlu, 2019). Therefore, metaphorical thinking has an effect on determining people’s perspectives, making sense of life, events, and situations, and reflecting everything around them in their minds (Pilav and Elkatmış, 2013). Since each person’s point of view and meaning will be different, a lot of metaphors arise about a situation. When comparing any two things, the use of metaphors is easy to show similarities between them or to increase their better comprehensibleness.

In this study, phenomenology was used from qualitative research patterns. Phenomenology is an appropriate method for examining cases where in-depth and detailed information about a situation is needed (Yıldırım and Şimşek, 2008, Neuman and Robson, 2014). Phenomenology is a describing research pattern and examines how participants make sense of perceptions and situations based on their individual experiences (Akturan and Esen, 2008).

The reason that metaphor analysis is preferred in this study is to take the experiences and perspectives of high school students in the pandemic process and the meanings they place on COVID-19 and the school in this process through metaphor and reveal the process behind the opinions.

### Research group

The research group was held in the 2020–2021 academic year with the participation of 134 Anatolian high school students studying in public schools in Hendek, district of Sakarya province.

In this research, a purposeful sampling was used. The purposeful sample attempts to obtain the desired information, and data collection is stopped if new information is not encountered (Coyne, 1997). In other words, the formation of a saturation point when the information begins to repeat can be considered as the first criterion (Lincoln and Guba, 1985). In phenomenological research, it is aimed to gain more information about the focusing phenomenon rather than the size of the sample, and the reason for this is to reach qualified and quality information (Sanders, 1982; Baş and Akturan, 2008; Patton, 2014). The demographic characteristics of the students in the study group and their families’ cases of COVID-19 are shown in Table 1.

**TABLE 1:** Demographics of participants.

Demographics features	Category	*n*	%
Gender	Female	92	68.7%
	Male	42	31.3%
Class level	9	39	29.1%
	10	25	18.7%
	11	41	30.6%
	12	29	21.6%
Does anyone in your family have COVID-19?	Yes	18	13.4%
	No	116	86.6%
Total		134	100%

According to Table 1, 68.7% (*n* = 92) female and 31.3% (*n* = 42) male high school students participated in the study. The distribution of students by class level is as follows: 29.1% are 9th graders (*n* = 39), 18.7% are 10th graders (*n* = 25), 30.6% are 11th graders (*n* = 41), and 21.6% are 12th graders (*n* = 29). About 13.4% (*n* = 18) of the responses to the question “Does anyone in your family have coronavirus?” answered yes, while a large majority, 86.6% (*n* = 116), answered no. With this question, it is also aimed to reflect the experiences of the people who experienced the phenomenon that was the subject of the research.

### Data collection tools

The data related to this research were collected with a questionnaire created electronically. The instruction for filling out the form has also been added to the form. When creating the questionnaire form, which is the data collection tool for the search, pre-made metaphor studies were examined (Saban, 2008; Erarsalan, 2011; Bozdaǧ and Dinç, 2020; Bozkurt, 2020). The final version has been given with expert opinion.

**FIGURE 1:**
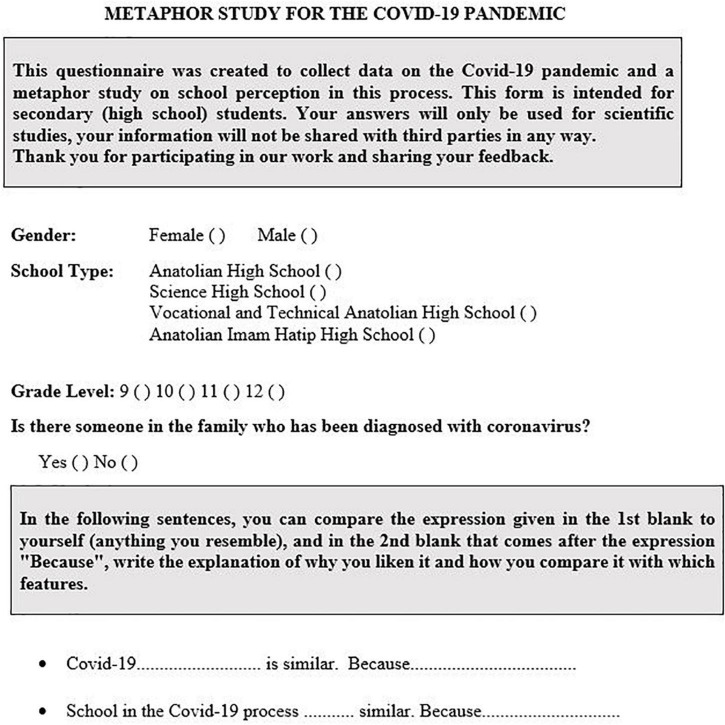
Data collection tool.

The data collection tool consists of two parts. In the first section, it is aimed to gather demographic information about the gender, school type, class level of the students, and whether there are individuals in the family who have been diagnosed with coronavirus. In the second part, “COVID-19…is similar. Because…” and “School in the COVID-19 process…similar. Because…” With these prepared sentences, students were asked to write only one metaphor in the specified spaces and to support these thoughts by explaining the reasons. By adding the concept of “because” to the research, students were asked to explain their own metaphors with a “justification” (or “logical basis”) (Saban, 2009). These statements, explained by the participants themselves, constituted the main data source of this research as a “document.” The data collection tool was given in Figure 1.

### Data collection process

The metaphorical perception form created in the research data was shared with the participants through online and the instruction with the necessary explanations for filling out the form was added to the form. The forms were shared with the participants on 27 November 2020 through teachers. In the online process, teachers were also given detailed information about the questions that students may receive about form or metaphors. The form filling process was terminated on 11 December 2020. The total time allocated for the collection of data is 15 days.

### Validity and reliability

In this research, long-term data collection, expert verification, and comparison of data were used within the scope of validity and reliability studies. Reliability according to Creswell (2003), from the perspective of the participants in the study, was expressed as ensuring the accuracy of the research findings. Maxwell (2008) proposed similar strategies to increase reliability in qualitative research. These are the ones that are going to condensed, long field data, rich data, participant verification, expert verification, data variation, semi-statistical information, and comparison (Yin, 2011). In factual studies to increase reliability, three of these strategies are often used intensively. These include data triangulation, member checking, and peer review. In the study, the results were tried to be reached by including the rich descriptions proposed by Lincoln and Guba (1985) for transferability studies. In addition, the process of collecting and analyzing data to ensure reliability is explained in detail.

The reliability of the research was represented by eight categories of metaphors created with COVID-19 and related metaphor images created under eight categories in the same way as the metaphors created for the school in this process. Different experts have been consulted on this issue. Accordingly, a list form with metaphors and categories was created and the expert faculty member in the same field was asked to match. During the research process, three independent researchers coded the analysis of the collected data. As a result of the coding, these researchers came together and expressed their opinions on the codes they reached. These independent researchers have several qualitative studies in the field of educational sciences. In this context, it is thought that they have sufficient experience in the creation of codes. By determining the number of consensus and disagreement in the pairings, the reliability of the research was calculated using Miles and Huberman (1994) formula (Reliability = consensus/consensus + consensus). Miles and Huberman (1994) stated that the intercoder consensus should be at least 70% according to the coding control that gives this ratio internal consistency. In this study, 79% of the results were obtained in the reliability study. In addition, the data used in the process of creating metaphors in the tables are shown by quoting directly.

### Analysis of data

In this research, the high school students were asked to produce metaphors about COVID-19 and their perception of the school in the process. “Content analysis” technique was used in the given analysis process. In the content analysis technique, similar information obtained in the research is put together under a heading that reflects this information, and categories are created. The organized data are interpreted understandably (Yıldırım and Şimşek, 2008; Büyüköztürk et al., 2011). In this research, the data were first examined, and forms that were not justified or associated with metaphors were determined and excluded. For the remaining 134 data after the eliminations, the forms of the students were numbered as P1, P2, P3, etc. The forms are listed individually, and the metaphors with similar meanings are combined and matched to the relevant categories based on the explanations made after “Because” in the category stage. Metaphors for each category are recurrence frequency, and percentage ratios are tabled and interpreted in the findings section.

In the study, COVID-19-related metaphors were collected in eight categories and school-related metaphors were collected in eight categories.

## Findings

In this section, the conceptual categories are created based on the metaphors produced by the participants regarding COVID-19 and the school in this process, and the explanations of these metaphors are presented in tables. When explaining the metaphors they produced, sample expressions of the participants were included. The findings obtained as a result of the analysis of the data are organized systematically and clearly.

### What are the metaphorical perceptions of high school students regarding “the COVID-19 pandemic”?

When the data were examined, it was seen that they produced 76 metaphors that were thought to be valid. The frequency and percentage of each metaphor were given by calculating how many students used the metaphor. The metaphors produced by the students for the COVID-19 pandemic and the frequency and percentage ratio of these metaphors are presented in Table 2.

**TABLE 2:** Metaphors created by students for the concept of “COVID-19.”

Metaphor sequence	Metaphor name	*f*	%	Metaphor sequence	Metaphor name	*f*	%
1	Flu	26	19.4%	39	Criminal crime	1	0.74%
2	Prison	6	4.5%	40	Cheetah	1	0.74%
3	Snake	6	4.5%	41	Gossip	1	0.74%
4	Fire	4	3%	42	Swine flu	1	0.74%
5	Human	4	3%	43	Enemy	1	0.74%
6	Bee	3	2.33%	44	Electric shock	1	0.74%
7	Tick	3	2.33%	45	Debris	1	0.74%
8	Insect	2	1.5%	46	Disaster	1	0.74%
9	Rotten fruit	2	1.5%	47	Football	1	0.74%
10	Earthquake	2	1.5%	48	Traveler	1	0.74%
11	Animal	2	1.5%	49	Race for life	1	0.74%
12	Winter	2	1.5%	50	Thief	1	0.74%
13	Germ	2	1.5%	51	Snoring	1	0.74%
14	Terminal illness	2	1.5%	52	Internet	1	0.74%
15	War	2	1.5%	53	Nightmare	1	0.74%
16	Exam	2	1.5%	54	Cage	1	0.74%
17	Cigarette	2	1.5%	55	Cancer	1	0.74%
18	Fly	2	1.5%	56	Capitalism	1	0.74%
19	Dust	2	1.5%	57	Characterlessness	1	0.74%
20	Poison	2	1.5%	58	Brother	1	0.74%
21	Justice	1	0.74%	59	Red line	1	0.74%
22	Lung blood pressure	1	0.74%	60	Dirt	1	0.74%
23	Relative	1	0.74%	61	Dog	1	0.74%
24	Scorpion	1	0.74%	62	Evil	1	0.74%
25	Allergic food	1	0.74%	63	Mine	1	0.74%
26	Alcoholic person	1	0.74%	64	Game	1	0.74%
27	Mother	1	0.74%	65	Spider	1	0.74%
28	Ancient Sparta	1	0.74%	66	Wind	1	0.74%
29	Acid rain	1	0.74%	67	Ivy	1	0.74%
30	Asthma	1	0.74%	68	Oil	1	0.74%
31	Love	1	0.74%	69	Magic	1	0.74%
32	Horse	1	0.74%	70	Chicken pox	1	0.74%
33	Separation	1	0.74%	71	Leech	1	0.74%
34	The Reaper	1	0.74%	72	Grape plant	1	0.74%
35	Puffer fish	1	0.74%	73	Loneliness	1	0.74%
36	Swamp	1	0.74%	74	Intensive care	1	0.74%
37	Bit	1	0.74%	75	Harmful disease	1	0.74%
38	Bomb	1	0.74%	76	Pneumonia	1	0.74%
Total						134	100%

When Table 2 is examined, they produced a total of 76 kinds of metaphors for the concept of “COVID-19” of high school students and stated a total of 134 opinions. The most repetitious metaphors produced by high school students regarding COVID-19, “flu” (*f* = 26), “Prison” (*f* = 6), and “Snake” (*f* = 6), were listed, while 56 metaphors were repeated once.

#### Categories created by high school students in terms of common characteristics of metaphors produced regarding the concept of “COVID-19”

When 76 different metaphors produced by the students were examined and evaluated, the metaphors were put together and categories were created based on the analogy aspects of the metaphors produced by the students. When the explanations of some metaphors were examined, it was seen that although the same metaphor was used, the relationship was established by justifying it in different ways. Due to this situation, they are in different categories. The metaphors created by the high school students on the concept of COVID-19 are collected in eight categories. These categories are “Disease,” “Animal,” “Damaging,” “Rapid Spread (spreading fast),” “Struggle,” “Restrictive,” “Responsibility,” and “Destruction.” The categories created from the metaphors produced by the students for the COVID-19 pandemic and the frequency and percentage ratio of these categories are presented in Table 3.

**TABLE 3:** Categories of metaphors related to the concept of “COVID-19” created by high school students.

Category	Metaphor	Metaphor type	Frequency (*f*)	Percent (%)
Disease	Flu (26), Cancer (1), asthma (1), Harmful disease (1), Pneumonia (1), Love (1), Swine flu (1), Chickenpox (1), Lung blood pressure (1)	9	34	25.4%
Animal	Snake (6), Puffer fish (1), Animal (2), Scorpion (1), Dog (1) Tick (3), Louse (1), Bee (3), Leech (1), Fly (2), Spider (1), Insect (2)	12	24	17.9%
Damaging	Cigarette (2), Germ (2), Poison (2), Dust (2), Acid rain (1), Electric shock (1), Evil (1), Rotten fruit (1), Human (3), Thief (1), Relative (1), Alcoholic person (1), Brother (1)	13	18	14.18%
Spreading fast	Fire (4), Ivy (1), Grape plant (1), Cheetah (1), Gossip (1), Snoring (1), characterlessness (1), Wind (1), Rotten fruit (1), Horse (1), Traveler (1), Oil (1),	12	16	11.94%
Struggle	Capitalism (1), Ancient Sparta (1), War (2), Intensive care (1), Red line (1), Winter (2), Game (1), Swamp (1), Grim reaper (1), Fatal (2), Cancer (1), Race for life (1), Enemy (1)	13	16	11.94%
Restrictive	Prison (6), Cage (1), Nightmare (1), Mother (1), Magic (1), Criminal offense (1), Separation (1), Loneliness (1)	8	13	9.7%
Responsibility	Exam (2), Justice (1), Dirt (1), Football (1), someone to learn from (1)	5	6	4.47%
Destruction	Debris (1), Disaster (1), Bomb (1), Mine (1), Earthquake (2)	5	6	4.47%
Total		77	134	100.00%

As shown in Table 3, metaphors created by the high school students were collected in eight conceptual categories according to their similarities. Since the “Human” metaphors mentioned in Table 2 were repeated in different categories due to the fact that they were in different categories due to the different distributions within themselves according to the explanations of the students after the expression “Because,” the total number of metaphor varieties increased to 77 in Table 3. The “disease” category was found to be the category in which metaphors were developed at the highest frequency. The categories created from the metaphors created by the students for the COVID-19 pandemic were explained, and sample expressions were given.

##### Category 1: “Disease”

The high school students who participated in the study produced the most metaphors in this category. The students expressed COVID-19 by associating it with similar symptoms, pandemics, or fatalities when explaining the metaphors in the disease category. The metaphors in the disease category are nine metaphors (flu, asthma, pneumonia, harmful disease, swine flu, fatal disease, chickenpox, lung blood pressure, and allergies) created by 34 students (25.4%). The most repetitious metaphor in this category is the metaphor “flu” (*f* = 26). Table 4 presents the metaphors in this category and the expressions of some students.

**TABLE 4:** Disease category.

Disease			
Metaphor	*f*	Participants (P)	Sample expressions
Flu	26	P5, P7, P13, P14, P15, P22, P28, P45, P46, P48 P58, P59, P61, P66, P69, P75, P77, P85, P88 P101, P109, P113, P115, P124, P125, P133	*“COVID-19 is similar to flu. Because the symptoms are the same and when you catch the flu they think you are COVID-19.” (P61)* *“COVID-19 is similar to flu. Because it shows the same symptoms.” (P14)* *“COVID-19 is similar to flu. Because there are symptoms of influenza.”*
Asthma	1	P91	*“COVID-19 is similar to a type of asthma. Because when we become COVID-19, we experience shortness of breath.” (P91)*
Swine flu	1	P38	*“COVID-19 is similar to swine flu. Because it’s an pandemic” (P38)*
Lung blood pressure	1	P119	“*COVID-19 is similar to lung blood pressure. Because its symptoms are the same.” (P119)*
Harmful disease	1	P29	
Pneumonia	1	P122	
Chickenpox	1	P63	
Allergic food	1	P3	
Love	1	P23	
Total	34		

##### Category 2: “Animal”

In the descriptions of the metaphors in this category, some participants of the metaphors were created for the COVID-19 pandemic; fear spoke of situations such as the lack of medication for the treatment and the fact that it carries deadly risks, while some participants associated it with the emphasis that it was barely removed from the body, and sickening and its effects would continue. This category consisted of 12 metaphor types created by 24 (17.9%) participants. The most recurring metaphor of the category is the “Snake” (*f* = 6) metaphor. Following this metaphor, Table 5 presents the metaphors in this category and the expressions of some students.

**TABLE 5:** Animal category.

Animal			
Metaphor	*f*	Participants (P)	Sample expressions
Snake	6	P12, P53, P82	*“COVID-19 is similar to a snake. Because without an antidote, many unconscious people can die.” (P110)*
		P106, P110, P131	*“COVID-19 is like a snake. Because it’s scary.”*
Bee	3	P55, P93, P129	*“COVID-19 is similar to a bee. Because when we do, we’ll get sick, maybe we’ll lose our lives.” (P55)*
Tick	3	P21, P65, P70	*“COVID-19 is similar to ticks. Because it’s sneaky and dangerous.” (P65)*
Animal	2	P40, P62	*“COVID-19 is similar to a predator. Because if we do, it could be dangerous for us.” (P40)*
Fly	2	P76, P102	*“COVID-19 is like a fly. Because it is a great one of the little devastations.” (P102)*
Insect	2	P108, P123	*“COVID-19 is like a pufferfish. Because it poisons the person it touches.” (P126)*
Puffer fish	1	P126	*“Similar to COVID-19 bite. Because once it’s infected, it’s hard to get out, so we have to take precautions.” (P73)*
Bit	1	P54	
Leech	1	P73	
Spider	1	P120	
Dog	1	P32	
Scorpion	1	P67	
Total	24		

##### Category 3: “Damaging”

In this category, the description of the metaphors with which COVID-19 is associated mentioned that it has a structure that harms the person and those around him and its infectious effects. The “Damaging” category includes six metaphors (human, cigarette, germ, poison, dust, evil, rotten fruit, thief, alcoholic person, electric shock, acid rain, relative, and brother) created by 16 students (14.18%). Table 6 contains metaphors and statements from some students in this category.

**TABLE 6:** Damaging category.

Damaging			
Metaphor	f	Participants (P)	Sample expression
Human	3	P6, P96	*“COVID-19 is like lying people because: when you believe in something, you tell everyone what you believe, and that’s how it goes.” (P98)*
		P98	*“COVID-19 is like a bad person. Because a bad person attacks everyone.” (P96)*
Germ	2	P8, P34	*“COVID-19 is similar to a microbe. Because when it enters the body, it hurts.” (P8)*
Cigarette	2	P2, P35	*“COVID-19 is similar to smoking. Because when you’re with the smokers, it hurts you, it does the same damage, and as a precaution, it’s important to stay away from the smoking environment.” (P2)*
Dust	2	P11, P92	*“COVID-19 is like evil. Because it’s so easy and deadly to spread.” (P107)*
Poison	2	P9	*“COVID-19 is like a thief. Because it stole our lives.” (P1)*
Thief	1	P1	*“COVID-19 is like fruit rot. Because if you put the rotten fruit next to the fresh one, the fresh one will rot.” (P18)*
Relative	1	P25	
Alcoholic person	1	P79	
Brother	1	P134	
Rotten fruit	1	P18	
Evil	1	P107	
Acid rain	1	P49	
Electric shock	1	P112	
Total	19		

##### Category 4: “Spreading fast”

In this category, students expressed the common feature of COVID-19 metaphors by associating them with fast growth and spreading, rapid movement, and easy adaptation to conditions. The category includes 12 metaphors (fire, ivy, grape plant, cheetah, gossip, snoring, characterlessness, wind, rotten fruit, horse, traveler, and oil) created by 16 students (11.94%). In Table 7, the metaphors in this category and the expressions of some students are given.

**TABLE 7:** Fast spanning category.

Spreading fast			
Metaphor	*f*	Participants (P)	Sample expressions
Fire	4	P27, P43	*“COVID-19 is like fire. Because you can’t intervene.” (P43)*
		P51, P56	*“COVID-19 is similar to fire. Because it burns where it touches.” (P56)*
Ivy	1	P16	*“COVID-19 is similar to ivy. Because it’s spreading more and more by the day and it’s all over the place.” (P16)*
Grape plant	1	P57	*“COVID-19 is similar to the Internet. Because it’s all over the world.” (P116)*
Cheetah	1	P19	*“COVID-19 is like gossip. Because it spreads and causes trouble to everyone.” (P33)*
Gossip	1	P33	*“COVID-19 is similar to fruit Rot.*
Snoring	1	P10	*Because it infects people with contact, such as the decay of a fruit that comes into contact with a rotten fruit.” (P114)*
Characterlessness	1	P30	
Wind	1	P89	
Internet	1	P116	
Rotten fruit	1	P114	
Horse	1	P60	
Traveler	1	P105	
Oil	1	P68	
Total	16		

##### Category 5: “The struggle”

In this category, the description of the metaphors associated with COVID-19 is explained on the basis of a struggle for life, difficult conditions, systemic structures, and the measures required by such periods. The “Struggle” category includes 13 metaphors (war, intensive care, red line, capitalism, ancient Sparta, winter, game, swamp, grim reaper, fatal, cancer, race for life, and enemy) created by 16 students (11.94%). In Table 8, metaphor distributions in this category and the expressions of some students are given.

**TABLE 8:** Struggle category.

Struggle			
Metaphor	*f*	Participants (P)	Sample expressions
Winter	2	P41, P104	*“COVID-19 is similar to a harsh winter. Because if we don’t take precautions, it will lead to serious deaths and destruction in the environment.” (P41)*
Fatal	2	P87, P117	*“COVID-19 is similar to a fatal virus. Because we can get sick and die.” (P87)*
Capitalism	1	P95	*“COVID-19 is similar*
Ancient Sparta	1	P99	*to capitalism. Because it destroys the weak.” (P95) “COVID-19 is like war. Because as the measure decreases, the losses multiply.” (P50)*
War	2	P20	*“It’s like an ICU.*
	1	P84	*Because we’ll either*
Red line	1	P44	*get out of intensive care alive or we’ll die.” (P84)*
Game	1	P36	*“COVID-19 is similar to the enemy. Because we’ll fight it.” (P81)*
Swamp	1	P130	
Grim reaper	1	P128	
Cancer	1	P90	
Race for life	1	P39	
Enemy	1	P81	
Total	16		

##### Category 6: “Restrictive”

In this category, the students tried to explain the metaphors they created for COVID-19 by associating them with the side that limits, restricts, and forces their lives to certain conditions. The “restrictive” category includes eight metaphors (prison, cage, nightmare, mother, magic, criminal crime, separation, and loneliness) created by 13 students (9.7%).

##### Category 7: “Responsibility”

In this category, the participants used the metaphors “Exam,” “Justice,” “Dirt,” “Football,” and “Someone to Learn from” to associate that there are rules for everywhere and situation, that attention should be paid, and that success will be achieved if action is taken knowing its responsibility. The six students (4.47%) produced these metaphors.

##### Category 8: “Destruction”

In this category, “Debris,” “Disaster,” “Bomb,” “Mine,” and “Earthquake” metaphors were created, while emphasizing the destructive effect of COVID-19, the negative emotions the students experience and the subsequent effects will continue and measures should be taken in the first place.

Table 9 contains metaphor distributions in this category and the expressions of some students.

**TABLE 9:** Restrictive category, responsibility category, and destruction category.

Category	Metaphor	*f*	Participant (P)	Sample expression
Restrictive	Prison	6	P31, P52, P83, P94	*“COVID-9 is like prison. Because it put us all in the house in the process, most of them took away our freedom.” (P94)*
	Cage	1	P42	*“COVID-19 is like prison. Because it traps you in the house.” (P52)*
	Nightmare	1	P24	*“COVID-19 is cage-like. Because it either kills people or it’s the scars of your life.” (P42)*
	Criminal crime	1	P71	*“COVID-19 is similar to separation. Because we are separated from everything and everyone.” (P103)*
	Separation	1	P103	
	Loneliness	1	P121	
	Magic	1	P47	
	Mother	1	P37	
	Total	13		
Responsibility	Exam	2	P4, P26	*“COVID-19 is similar to an exam. Because you need to be instantly careful with the exam so as not to make any mistakes.” (P4)*
	Justice	1	P74	*“COVID-19 is like justice. Because it punishes those who do not follow the rules.” (P74)*
	Dirt	1	P97	*“COVID-19 is like someone to learn from. Because it reminds us of some of the responsibilities we have to take.” (P132)*
	Football	1	P86	
	Someone to learn from	1	P132	
	Total	6		
Destruction	Earthquake	2	P72, P111	*“COVID-19 is similar to an earthquake. Because if you don’t take precautions, it could lead to major disasters.” (P72)*
	Debris	1	P17	*“COVID-19 is like a disaster. Because it turned everything upside down.” (P64)*
	Disaster	1	P64	*“COVID-19 is similar to mine. Because if you step on a mine you put yourself and those around you in danger. (P118)*
	Bomb	1	P78	
	Mine	1	P118	
	Total	6		

### What are the metaphorical perceptions of high school students regarding “school” during the COVID-19 pandemic?

When the data were examined, 78 metaphors thought to be valid were produced. The frequency and percentage ratio of each metaphor were given by calculating the number of students who produced the same metaphor. The metaphors produced by the students for the school during the COVID-19 process and the frequency and percentage of these metaphors are included in Table 10.

**TABLE 10:** Metaphors created by the students for the concept of “school in this process.”

Metaphor number	Metaphor name	*f*	%	Metaphor number	Metaphor name	*f*	%
1	Hospital	11	8.2%	40	Computer without Internet	1	0.75%
2	Abandoned place	10	7.5%	41	The place where you look blankly on the Internet	1	0.75%
3	Free space	6	4.5%	42	Suicide	1	0.75%
4	Operating room	4	3%	43	To work	1	0.75%
5	Factory	4	3%	44	Torture	1	0.75%
6	Construction site	4	3%	45	Shield	1	0.75%
7	Holiday	4	3%	46	Closed shop	1	0.75%
8	Home	3	2.31%	47	Fly	1	0.75%
9	Laboratory	3	2.31%	48	Quarantine	1	0.75%
10	Mined field	3	2.31%	49	A place where no one lives	1	0.75%
11	Game	3	2.31%	50	Book	1	0.75%
12	The battlefield	3	2.31%	51	Bucket	1	0.75%
13	Cycling-riding motorcycles	2	1.5%	52	Resentment	1	0.75%
14	Bomb	2	1.5%	53	Maze	1	0.75%
15	Space	2	1.5%	54	Armageddon	1	0.75%
16	Contamination center	2	1.5%	55	Germ nest	1	0.75%
17	Prison	2	1.5%	56	Kitchen	1	0.75%
18	Winter	2	1.5%	57	Rusted iron	1	0.75%
19	House of horror	2	1.5%	58	Homing pigeon	1	0.75%
20	Marketplace	2	1.5%	59	Russian roulette	1	0.75%
21	Hazardous area	2	1.5%	60	Fighting soldier	1	0.75%
22	Restricted area	1	0.75%	61	Beloved film	1	0.75%
23	Open education	1	0.75%	62	Street	1	0.75%
24	Shopping mall	1	0.75%	63	Social media	1	0.75%
25	Bank queue	1	0.75%	64	Place of socialization	1	0.75%
26	Empty lesson	1	0.75%	65	Public transportation	1	0.75%
27	Empty tin	1	0.75%	66	Distant relative	1	0.75%
28	Heaven	1	0.75%	67	Long distance relationship	1	0.75%
29	Child’s toy	1	0.75%	68	Space	1	0.75%
30	Parent who lost children	1	0.75%	69	Exists but does not exist	1	0.75%
31	Dump	1	0.75%	70	Virus house	1	0.75%
32	Education	1	0.75%	71	Foreign language	1	0.75%
33	Safety area	1	0.75%	72	Volcano	1	0.75%
34	Universe	1	0.75%	73	To old age	1	0.75%
35	Dream	1	0.75%	74	Inadequacy	1	0.75%
36	Uninhabited island	1	0.75%	75	Intensive care	1	0.75%
37	Desolate house	1	0.75%	76	YouTube video	1	0.75%
38	Deserted	1	0.75%	77	Harmful place	1	0.75%
39	Hollow	1	0.75%	78	Challenging task	1	0.75%
Total						134	100%

When Table 10 is examined, the students produced a total of 78 kinds of metaphors for the concept of “School in the COVID-19 Process” and stated a total of 134 opinions. The top three most repetitive metaphors produced about school during the COVID-19 process by high school students were “Hospital” (*f* = 11), “Abandoned (place, house, mansion, building, and space)” (*f* = 10), and “Free space (building, playground, warehouse, and street)” (*f* = 6). Fifty-seven metaphors observed were repeated once.

#### Categories created by high school students in terms of common characteristics of metaphors produced for the concept of “school in the COVID-19 process”

Metaphors created by high school students were collected in eight conceptual categories according to their similarities. These categories are “Risky Area,” “Lack of Complementary Elements,” “Place to Take Precautions,” “Dysfunctional,” “Continuity in Education,” “Challenging Educational Activities,” “Social Relationship,” and “Safe Environment.” Metaphor distributions of this category are presented in Table 11.

**TABLE 11:** Categories of metaphors created by high school students regarding the concept of “school during COVID-19.”

Category	Metaphor	Type of metaphor	Frequency (*f*)	Percent (%)
Risky area	Mined field (3), Contamination center (2), Hospital (2), House of horror (2), Bomb (2), Battlefield (2), Restricted area (2), Marketplace (2), Hazardous area (2), Factory (1), Russian roulette (1), Suicide (1), Harmful place (1), Volcano (1), Bank queue (1), Virus House (1), Street (1), Quarantine (1), Shopping mall (1), Blackfly (1), Public transportation (1), Germ nest (1), Dump (1)	20	33	24.63%
Lack of complementary elements	Abandoned (place, house, mansion, building, space, factory) (11), Free space (building, playground, warehouse, street, factory) (7), Space (1), A place where no one lives (1), Uninhabited (place, island, house) (3), A parent who lost children (1), Closed shop (1), Buck (1), Hollow nut (1), Intensive care (1), A computer without Internet (1)	11	29	21.64%
Place to take precautions	Hospital (9), Construction site (4), Operating room (3), Laboratory (3), Winter (2), Book (1), Office (1), Factory (1), Battlefield (1), Heaven (1), Cycling (1), Riding motorcycle (1), Kitchen (1)	13	29	21.64%
Dysfunctional	Holiday (4), Space (2), Empty tin (1), Dream (1), Exists but does not exist (1), Inadequacy (1), Empty lesson (1), Universe (1), Rusted iron (1), The place where you look blankly on the Internet (1)	10	14	10.45%
Continuity in education	Home (2), Armageddon (1), Maze (1), Education (1), Fighting soldiers (1), Old age (1), Open education (1), YouTube videos (1), Homing pigeon (1), Social media (1), Game (1)	11	13	9.7%
Challenging educational activities	Prison (2), Child’s toy (1), Game (1), Foreign language (1), Challenging Mission (1), Torture (1)	6	7	5.22%
Social relationship	Long distance relationship (1), Resentment (1), Socializing place (1), Beloved film (1), Socializing place (1), Distant relative (1), Beloved film (1)	5	5	3.73%
Safe environment	Home (1), Shield (1), Safety place (1), Operating room (1)	4	4	2.99%
Total		80	134	100%

As seen in Table 11, the “Risk Area” category (*f* = 33) was the most intense category in frequency. This is followed by the categories “Lack of Complementary Elements” (*f* = 29) and “Where to take Precautions” (*f* = 29). According to the explanations of the students after the expression “Because,” the metaphors of “Human,” “Factory,” and “Operating Room” mentioned in Table 12, they showed different distribution within themselves and took place in different categories. Since it is repeated in the categories resulting from this, the total number of metaphor varieties in Table 13 shows 80. The conceptual categories of metaphors are discussed separately.

**TABLE 12:** Risky area category.

Risky area			
Metaphor	*f*	Participants (P)	Sample expressions
Mined field	3	P14, P86, P118	*“A school is similar to a mined field during COVID-19. Because if we are not cautious, we could die.” (P86)*
Contamination center	2	P2, P87	*“During COVID-19, the school is similar to the bomb that exploded. Because students don’t pay much attention and help spread it more quickly.” (P54)*
Hospital	2	P8, P119	*“In the COVID-19 process, the school is similar to a house of horrors. Because no student can go there with peace of mind. They’re going to have this constant fear of “What if I get it?”” (P95)*
Bomb	2	P54, P58	*“In the COVID-19 process, the school is similar to the market. Because families can be carriers and infect other families.” (P43)*
House of horror	2	P19, P95	
Battle field	2	P20, P96	
Restricted area	2	P39, P64	
Market	2	P43, P63	
Hazardous area	2	P121, P132	
Dump	1	P18	*“During the COVID-19 process, the school is similar to the bank queue. Because if too many people go down, the virus could spread.” (P47)*
Suicide	1	P25	*“In the COVID-19 process, the school is similar to the street. Because it’s an unsafe environment.” (P123)*
Harmful place	1	P29	*“In the COVID-19 process, the school is similar to blacks. Because just as blacks collect germs from wherever they are placed and deliver them to humans, they can collect germs from anywhere in school and these microbes and bacteria can reach us. “(P100)*
Volcano	1	P33	
Bank queue	1	P47	
Virus house	1	P122	
Street	1	P123	
Quarantine	1	P124	
Factory	1	P50	
Shopping mall	1	P75	
Fly	1	P100	
Public transportation	1	P102	
Russian roulette	1	P101	
Germ nest	1	P6	
Total	34		

**TABLE 13:** Lack of complementary element category.

Lack of complementary elements			
Metaphor	*f*	Participants (P)	Sample expressions
Abandoned (place, house, mansion, building, space, factory)	11	P30, P34, P45, P52, P62, P70, P81, P103, P115, P133, P134	*“During COVID-19, the school is like an abandoned place. Because there’s no one in schools.”(P52)*
Free space (building, playground, warehouse, street, factory)	7	P55, P61, P65 P67, P94, P117, P129	*“During COVID-19, the school is like an abandoned place. Because there are no students.” (P62)*
Uninhabited (place, island, house)	3	P13, P22, P89	*“During COVID-19, the school is like an empty playground. Because they’re united in the same denominator, both sad and empty.” (P65)*
Space	1	P9	*“During COVID-19, the school is like an empty warehouse. Because right now, not all schools have any hollow students or teachers.” (P129)*
A place where no one lives	1	P15	*“In the COVID-19 process, the school is similar to a deserted house. Because that’s where no one can go.” (P89)*
The parents who lost their children	1	P27	*“During COVID-19, the school is similar to a desert island. Because there are few.” (P22)*
Closed shop	1	P85	*“During COVID-19, the school is similar to parents who lost their school children, because school has lost its students who are what make the school.” (P27)*
Buck	1	P83	*“During COVID-19, the school is similar to the intensive care unit of the hospital. Because it’s desperation.” (P12)*
Hollow nut	1	P112	*“During COVID-19, the school is similar to a computer without the Internet. Because just like a computer is nothing without Internet, we’re never students at school.” (P120)*
Intensive care	1	P120	
A computer without Internet	1	P120	
Total	29		

##### Category 1: “Risky area”

High school students who participated in the study created the most metaphors in this category. In the “Risky Area” category, participants explained their metaphors for school during COVID-19 by associating them with a high risk of disease transmission. This category covers the most types of metaphors, including 20 metaphors (contamination center, hospital, mined field, landfill, horror house, suicide, harmful place, battlefield, volcano, restricted area, market, factory, bomb, Russian roulette, bank queue, hazardous area, virus house, street, and quarantine) created by 33 students (24.63%). Table 12 presents the distribution of metaphors and some selected student expressions in this category.

##### Category 2: “Lack of complementary elements”

In this category, it is seen that the participants create metaphors which emphasize the school exists only with its students and that no one is in the school in this process. These metaphors are abandoned, empty space, desolate, space, where no one lives, parents who have lost their children, closing shop, hollow nuts, buckets, intensive care, and computer without Internet. The codes in this category were created by 29 students (21.64%). The metaphor “Abandoned” (*f* = 11) was the most used metaphor by the students. Table 13 includes the distribution of metaphors and some student statements.

##### Category 3: “Place to take precautions”

In this category, students expressed the important place to take precautions in the explanations of the metaphor created for the school during the COVID-19 process. This category includes 13 kinds of metaphors (hospital, winter, construction site, operating room, book, factory, workplace, paradise, laboratory, battlefield, cycling, motorbike riding, and kitchen) created by 29 students (21.64%). Table 14 includes the distribution of metaphors and some student statements.

**TABLE 14:** Take precaution place category.

The place to take precaution			
Metaphor	*f*	Participants (P)	Sample expressions
Hospital	9	P3, P11, P28, P73, P88, P97 P104, P108, P38	*“In the COVID-19 process, the school is similar to the hospital. Because school is similar to a hospital, the disease can be transmitted, so we should wear masks and use disinfectant.” (P_3_)*
Construction site	4	P7, P76 P77, P113	*“In the COVID-19 process, the school is similar to the hospital because we distance ourselves from people and try not to get sick from them.” (P11)*
Operating room	3	P31, P46, P57	*“In the COVID-19 process, the school is similar to the hospital. Because all precautions are taken in the hospital.” (P104)*
Laboratory	3	P40, P80, P105	*“The school is similar to construction site during the COVID-19 process. Because just as we use helmet steel shoes in the construction area, we should use mask disinfectant in school and pay attention to social distancing and hand cleaning.” (P76)*
Winter	2	P4	*“The school is similar to construction site during the COVID-19 process. Because certain measures should be taken, such as in construction.” (P77)*
Book	1	P32	*“The school is like an operating room. Because we have to use certain things like masks and gloves.” (P_31_)*
Riding a bike	1	P79	*“During the COVID-19 process, the school is similar to the laboratory. Because we have to follow the rules so as not to get hurt.” (P40)*
The battlefield	1	P91	*“In the COVID-19 process, school is similar to winter. Because in winter, we don’t go out without coats, boots, scarves, and in the process, you don’t go out without a mask, without disinfectant.” (P4)*
Factory	1	P106	*“During the COVID-19 process, the school is like a battlefield. If you don’t get the ingredients, the virus will beat you.” (P91)*
Riding motorcycle	1	P110	
Kitchen	1	P131	
Heaven	1	P1	
Office	1	P90	
Total	29		

##### Category 4: “Dysfunctional”

Looking at the similarities of the metaphors in this category, the participants emphasize that the school has lost its function in this process and has not added anything to them and that a long-term break has been given. The category includes 10 types of metaphors (vacation, space, empty tin, dream, existing but not present, inadequacy, empty lesson, universe, rusted iron, and place to stare blankly over the Internet) created by 14 students (10.45%). Table 15 includes the distribution of metaphors and sample student expressions.

**TABLE 15:** Categories are dysfunctional, continuity in education, challenging educational activities, social relationship, and safe environment.

Category	Metaphor	*f*	Participants (P)	Sample expressions
Dysfunctional	Dysfunctional	4	P48, P66, P68, P107	*“During COVID-19, school is similar to a 3-month holiday. Because if we were in distance education, we wouldn’t get our full education.” (P107)*
	Space	2	P24	*“During COVID-19, school is like a break. Because everyone is in holiday mode at home.” (P68)*
	Empty tin	1	P41	*“During COVID-19, the school is similar to an empty lesson. Because we’ll have fun, but we’ll be untrained.” (P51)*
	Dream	1	P42	*“School shortages are similar in the COVID-19 process. Because no one understands anything, a process in which 3 people in 1 house without tablets cannot attend class at the same time, mothers are sad and helpless.” (P82)*
	Exist but not exist	1	P44	*“During the COVID-19 process, the school is like an empty tin. Because when you hit it, it sounds like it’s full, but there’s nothing useful about it. It’s empty-headed ironing.” (P41)*
	Empty lesson	1	P51	
	Universe	1	P53	
	Rusted iron	1	P72	
	Inadequacy	1	P82	
	A place to stare blankly over the Internet	1	P116	
	Total	14		
Continuity in education	Home	2	P69, P127	*“The school is similar to home during the COVID-19 process. Because you can continue your education from home.” (P127)*
	Game	2	P99, P114	*“The school is similar to game during the COVID-19 process. Because the more active you are and the more you work, the more you earn.” (P114)*
	Old age	1	P130	*“In the COVID-19 process, the school is similar to a maze. Because we never get out of it, and the lessons go on.” (P56)*
	Fighting soldier	1	P17	*“During the COVID-19 process, the school is similar to the soldiers who fought. Because they are fighting for homeland (education) in one of the places where there is a high risk of viruses.” (P17)*
	Open education	1	P5	*“During the COVID-19 process, the school is similar to education Because if we continue in the process and not be careful, the consequences can upset us all.” (P59)*
	YouTube videos	1	P92	
	Social media	1	P26	
	Apocalypse	1	P49	
	Maze	1	P56	
	Education	1	P59	
	Homing pigeon	1	P126	
	Total	13		
Challenging educational activities	Prison	2	P10, P21	*“In the COVID-19 process, the school is similar to prison. Because it condemns us to technological instruments.” (P21)*
	Child’s toy	1	P16	*“During COVID-19, School is similar to playing doom game in a hard mood. Because it makes everything difficult. For example, placing Physical Education and Sports lessons to online education schedule.”(P60)*
	Foreign language	1	P74	*“In the COVID-19 process, school is like torture. Because we get up early in the morning and do lessons and homework till nightfall.” (P128)*
	Game	1	P60	
	Challenging task	1	P109	
	Torture	1	P128	
	Total	7		
Social relationship	Resentment	1	P125	*“In the COVID-19 process, the school is similar to resentment. Because the school is closed, there’s always a distance between us and our friends.” (P84)*
	Distant relative	1	P111	*“In the COVID-19 process, the school is similar to the place of socialization. Because we couldn’t get out of the house, but we used to socialize at school.” (P125)*
	The movie we like	1	P71	*“During the COVID-19 process, the school is similar to your distant relative. Because you’re going to miss it right now, but you can’t go.” (P111)*
	Place of socialization	1	P10	
	Long distance relationship	1	P23	
	Total	5		
Safe environment	Shield	1	P78	*“During the COVID-19 process, the school is similar to safety place. Because school is the most sheltered place.” (P98)*
	Safety place	1	P98	*“During COVID-19, the school is similar to a shield. Because it protects us.” (P78)*
	Operating room	1	P36	*“The school is similar to our home during the COVID-19 process. Because a safe environment is created.” (P35)*
	Our house	1	P35	
	Total	4		

##### Category 5: “Continuity in education”

The “Continuity in Education” category is also a category in which metaphors are created based on the educational aspect of the school. The metaphors created in this category emphasized the school’s non-suspension of education and the retention of students in education, the continuation of home education, and the way and how the course is processed. The distribution of metaphors and student expressions are presented in Table 15 for this category consisting of 11 types of metaphors and 13 students (9.7%), including “Apocalypse,” “Maze,” “Education,” “Home,” “Game,” “Old Age,” “Fighting Soldier,” “Open Education,” “YouTube Videos,” “Homing Pigeon,” and “Social Media.”

##### Category 6: “Challenging educational activities”

In this category, participants consider the school in terms of educational activities during the COVID-19 process. They associated the impact of the school’s responsibilities with the six metaphors they created (prison, child’s play, play, foreign language, challenging duty, and torture). Seven (5.22%) students were included in the category. In Table 15, metaphor distributions and expressions of the participants are included.

##### Category 7: “Social relationship”

In the “Social Relationship” category, participants discussed the social side of the school with the metaphor they used. They treated it as a structure in which they established social relations in school and brought them together. This category includes five types of metaphors (long-distance relationship, resentment, distant relative, favorite film, and place of socialization) created by five students (3.73%). Table 15 contains metaphor distributions and some example expressions in this category.

##### Category 8: “Safe environment”

Four of the students (2.99%) who participated in the study see the school as a safe place where the measures taken are applied. “Home,” “Shield,” “Operating Room,” and “Safety place” metaphors were used. Table 15 contains metaphor distributions and student expressions.

## Discussion and conclusion

This research was carried out to reveal the perspectives of high school students regarding the COVID-19 pandemic and the concept of school through their metaphors. The COVID-19 pandemic, which has affected our country and started to spread rapidly, has created a new experience in the educational lives of the students by bringing some innovations within the scope of the measures in the field of education. The concept of the COVID-19 pandemic and schools in this process was thought to have different meanings according to the perspectives of the students.

The high school students developed a total of 76 metaphors for the concept of “COVID-19 pandemic” and expressed 134 opinions. Twenty metaphors showed a frequency of repetition between 26 and 2, while 56 metaphors were used once. The large number of metaphors repeated once indicates the differences in individual perception of the COVID-19 pandemic and the approach to the situation from different perspectives. When the most repeated metaphors of the students were examined, metaphors such as Flu, Prison, Snake, Fire, and Human were determined.

In the “Disease” category in the study, metaphors such as flu, asthma, swine flu, and lung blood pressure were produced. These metaphors showed that COVID-19 disease is similar to some diseases in people showing symptoms such as fire, fatigue, weakness, cough, and muscle aches. In this respect, according to the World Health Organization [WHO] (2020) report, the most common symptoms of the COVID-19 pandemic are fever, cough, weakness, shortness of breath, and a small number of diarrhea. Therefore, the students have similarities to the COVID-19 pandemic based on the same symptoms. The metaphor for lung blood pressure in this category, due to the damage to the lung, is thought to be identified with the impact of the COVID-19 pandemic. In their study examining the perceptions of secondary school students regarding COVID-19, Görgülü Arı and Arslan (2020) concluded that the COVID-19 pandemic is similar to the diseases known and encountered in daily life.

The most repeated metaphors in the “Animal” category are snake, bee, and tick metaphors. Some of the animal metaphors in this category are scary and dangerous and some are similar to COVID-19, based on the sticky, sneaky, and the damage it causes. When the metaphors are examined here, it can be said that unconscious movement carries a fatal risk, and media reports and opinion experts have gained such a place in the perception of the students.

Some of the metaphors in the “Damaging” category are cigarettes, fruit rot, man, and thief metaphors. With these metaphors, they have identified the effects of COVID-19 both on the person himself and on the people around him. It is thought that a person with the disease will have a domino effect if they are in the same environment as healthy people. At the same time, there is a perception in students that they are aggressive and negatively changed their life conditions.

In the “Spreading Fast” category, metaphors such as fire, ivy, cheetah, gossip, and traveler were created. With these metaphors, it is stated that COVID-19 disease is transmitted and adapts easily to fast-moving and growing conditions. Student metaphors are supported by the fact that COVID-19 has taken over the world in a short time. As a result of the investigation of the COVID-19 pandemic, it has been determined that the transition between people and its spread through objects is the most important feature of the pandemic (Karcıoǧlu, 2020). In addition, similar metaphors were categorized under the theme of “Contagion” in the study in which middle school students examined their perceptions of COVID-19 (Görgülü Arı and Arslan, 2020). It can be said that similar perceptions occur regardless of the level of the students.

When the “Struggle” category was evaluated in the study, it was determined that the COVID-19 pandemic was similar to the metaphors that it was a struggle in all respects. Metaphors such as winter, deadly, capitalism, war, and race for life were produced. It is not just emphasized that it carries a fatal risk. It can be said that the students have the perception that the disease does not separate age groups, and at the end of the process, the strong can survive in all respects. Karcıoǧlu (2020) stated in the COVID-19 study that the pandemic caused a lot of damage and death compared to SARS and MERS diseases. It supports the metaphors produced by the students in the struggle category in this respect.

The most repetitive metaphor in the “Restrictive” category is prison. The metaphors that follow are cages, nightmares, criminal offenses, separation, and loneliness. With these metaphors, the students stated that their lives were restricted by the pandemic, communication with their environment decreased or decreased and they stayed away from social life. Within the scope of the measures taken by the Ministry of Health to prevent the spread of the pandemic during the COVID-19 process, places that are dense in people for certain periods of time and where there will be difficulty in implementing distance rules have been temporarily closed. In addition, curfews covering certain age groups and weekends have been introduced. In this process, students were seen to express themselves using metaphors that isolated them from life. The mention of the same conclusions under the category of “Social Distancing” in the study on COVID-19 perception can be shown as evidence of this situation (Görgülü Arı and Arslan, 2020).

In the “Responsibility” category, metaphors such as exams, justice, and someone to be taught were used in the research. With these metaphors, it is seen that it integrates with the need to reveal the feelings of responsibility that societies should have in pandemic processes. It was seen here as a structure with COVID-19 rules. There is a perception in students that there will be negative consequences if the rules are not followed.

Looking at the literature, as a metaphorical study for the COVID-19 pandemic, it gathered under 45 different metaphors and six categories with 100 students participating in the “Metaphorical Perceptions of Middle School Students for COVID-19” studies carried out by Görgülü Arı and Arslan (2020). These categories are designated as “Infectious, Fatal, Socially Distancing, Disease-Causing, Late Noticed, and Related to Other Diseases.” The categories associated with “Contagion,” “Social Media Removal,” and “Other Diseases” and the “Rapid Spreading,” “Restrictive,” and “Disease” categories, respectively, are similar and reflect almost the same result. Looking at the “Fatal” category, the metaphors and justifications used in the category “Harmer,” “Animal,” and “Struggle” are similar in this study. The metaphor of “louse,” which is commonly seen in both studies, was used for stating its ability to pass from one person to another by Görgülü Arı and Arslan (2020), while the students in this study associated it as a problem. The metaphor “earthquake” is a metaphor used in both studies and expressed as causing destruction.

When we looked at the findings of the students’ perceptions of the “school” during the COVID-19 process, it was determined that the high school students produced a total of 78 metaphors for this concept and expressed 134 opinions. While 21 types of metaphors showed a frequency of repetition between 11 and 2, 57 metaphors were found to have been used once. The high number of repetitor metaphors indicates that they have various individual perspectives on the concept of school during the COVID-19 process. The most frequent metaphors of the students were determined as hospital, abandoned, and empty space metaphors, respectively.

In the “Risky Area” category in the research, metaphors such as mined field, contamination center, bomb, and house of horrors have been produced. They stated that these metaphors are similar to the concept of school in the COVID-19 process as a place considered risky in terms of the spread of the pandemic. Media reports and warnings by the health ministry to stay away from crowded environments create such a perception in the minds of students. In addition, the COVID-19 study found that it is easily spread among humans and that the virus can remain on surfaces for a certain period of time (Karcıoǧlu, 2020). This supports students to consider the school risky in this process. In addition, evaluating the perceptions of students who are having education in “health” regarding the COVID-19 pandemic, the study concluded that students had high concerns about the spread of the virus (Arslan and Filiz, 2020).

The research includes metaphors such as “Lack of Complementary Elements”: abandoned, empty space, desolate, space, closing shop, and parent who has lost children. In this process, the metaphors associated with the fact that the school is far from the students who are the most important complementary subject are noted. With the extension of the suspension of education in schools within the scope of the measures in March, it is seen that it affects the perceptions about the school by the students in this direction.

In the category of “The place to take precaution,” by using metaphors such as hospital, construction site, operating room, and laboratory, students point out that school is an area where attention should be paid to the COVID-19 process and sensitivity should be shown in complying with the rules. In producing these metaphors, it can be said that the students commented on the decision of the schools to open on 4 January 2021 and had the perception of continuity of the precautionary rules in the school after it was opened. In the same way, Arslan and Filiz (2020) concluded that the students are aware of compliance with the measures taken by the state and fulfilling their individual responsibilities in their studies on COVID-19 perceptions.

In the “Dysfunctional” category, the metaphors such as holidays, gaps, empty lessons, inadequacy, and empty tins were used and the school is addressed in terms of “education.” In this process, online educational activities carried out by students are considered dysfunctional and inadequate. With the closure of the schools, the efforts to continue educational activities brought the necessity of having the technology and the necessary skills to use it effectively. Niemi and Kousa (2020) conducted a study of teacher and student perceptions during the COVID-19 pandemic, stating that the students had negative perceptions about the learning methods brought about by distance education and that the problems caused by technological deficiencies resulted in inefficiencies in educational activities in this period. Bozkurt (2020) examined the perceptions of primary school students for distance education during the COVID-19 process, and although the metaphors produced in the category of inadequacy were different, they reached a conclusion in line with this study that negative perceptions of distance education exist and distance education is not suitable for everyone.

In the “Continuity in Education” category, the concept of school in the COVID-19 process was seen as an institution that continued the educational activities no matter how challenging the process is, by using metaphors such as home, fighting soldier, open education, and social media maze. The execution of the lessons and the teaching methods used are identified with digital platforms. The Ministry of Education has tried to widen the opportunity of students to reach educational activities and materials by developing the Education Information Network (EBA) platform as well as television lessons. It is seen that continuing the education process on television and web basis affects the perceptions of the students toward the school.

Another category of “Challenging Educational Activities” category, which includes metaphors related to the educational aspect of the school, includes metaphors such as prison, torture, and challenging missions. Participant students stated that during the COVID-19 process, the school forced students to use technological tools intensively, complicated educational activities, and increased their responsibilities (homework, lessons).

The category “Social Relations” includes metaphors such as resentment, distant relative, and place of socialization. The school is seen by students as a structure that enables interaction with its social environment. It can be said that the closure of schools during COVID-19 has led students to a negative sense of perception and loneliness. Bozkurt et al. (2020) stated that the students who stayed away from the school during the pandemic process were not only subjected to an educational interruption, but also stayed away from the social communication and interactions provided by the school.

The “Safe Environment” category is the only category in which completely positive perceptions are reflected in all of the metaphors used. The metaphors used are shield, safety place, operating room, and home. For students, the school has been found to be a safe environment where measures have been implemented during the COVID-19 process and negativity has not occurred.

There are no metaphor studies related to the school during the COVID-19 process. However, there have been previous studies with school perception. One of these studies, conducted by Özdemir (2013) on school perception and school commitment, concluded that the positive perceptions of students toward the school were moderately intense. In another study by Özdemir (2012) which investigates the school perception of high school students, the study concluded that while the school perceptions of students in the lower income group and freshmen were positive, those of students in the upper-income level and upper classes have negative perceptions. However, since the benefits of the COVID-19 process provide a new life on school experiences, it is not comparable to these studies.

## Recommendations

According to the results of the research, both the COVID-19 pandemic and the negative perceptions of the school should be tried to be eliminated in this process. For this purpose, support activities can be carried out to facilitate this process with the help of experts in the field of psychological counseling.

It should be explained that students can easily get through this process as long as they follow the necessary measures within educational activities. It should be stated that the rules and measures put in place for adverse sensory situations that can be created by the perception that there is an infectious and deadly risk are applicable and can be protected from the virus with a few very simple measures.

It would be appropriate for teachers and administrators at school to use more positive concepts or metaphors when talking about COVID-19. In this way, very negative perceptions against COVID-19 formed in the minds of students can be reduced to some extent.

Scientific studies on COVID-19 treatments aimed at reducing students’ anxiety can be mentioned. In addition, the details of the study in the field to improve COVID-19 can be mentioned.

With face-to-face training, it may be recommended to integrate remote learning environments into training programs as part of the training.

In the studies to be carried out regarding the COVID-19 pandemic, it can be recommended to examine the metaphor perceptions of the students regarding the school and the metaphor perceptions of the process as a whole. In this way, examining the changes in the perceptions of the students in the process of the COVID-19 pandemic and taking measures against them will accelerate. In addition, it is essential to carry out similar metaphorical studies at all education levels (preschool, primary education, secondary education, and university) to understand the process entirely. However, similar studies can be conducted with all education stakeholders (parents, teachers, students, and administrators). Thus, since the process will be thoroughly evaluated from the perspective of all stakeholders, the perceptions caused by the COVID-19 education process will be better understood.

## Data availability statement

The original contributions presented in this study are included in the article/Supplementary material, further inquiries can be directed to the corresponding author.

## Ethics statement

The studies involving human participants were reviewed and approved by the Ethics Committee of Sakarya University, following the guidelines of the Ethics Committee (E-61923333-050.99-13624). The participants provided their written informed consent to participate in this study.

## Author contributions

ÖV and AK contributed to conception and design of the study. MB organized the database. ÖV and ZD performed the coding of the collected data. AK wrote the first draft of the manuscript. AK, CB, ZD, and MB wrote sections of the manuscript. All authors contributed to manuscript revision, read, and approved the submitted version.
